# Therapeutic drug monitoring of nevirapine in saliva in Uganda using high performance liquid chromatography and a low cost thin-layer chromatography technique

**DOI:** 10.1186/1471-2334-14-473

**Published:** 2014-09-01

**Authors:** Mohammed Lamorde, Quirine Fillekes, Kim Sigaloff, Cissy Kityo, Allan Buzibye, Joshua Kayiwa, Concepta Merry, Lillian Nakatudde-Katumba, David Burger, Tobias F Rinke de Wit

**Affiliations:** Infectious Diseases Institute, Makerere University College of Health Sciences, Kampala, Uganda; Department of Pharmacy, Radboud University Medical Centre, Nijmegen, The Netherlands; Department of Global Health and Department of Internal Medicine; Academic Medical Center, University of Amsterdam; Amsterdam Institute for Global Health and Development, Amsterdam, The Netherlands; Joint Clinical Research Centre, Kampala, Uganda; Trinity College Dublin, Dublin, Ireland; Northwestern University, Chicago, USA

## Abstract

**Background:**

In resource limited settings access to laboratory monitoring of HIV treatment is limited and therapeutic drug monitoring is generally unavailable. This study aimed to evaluate nevirapine concentrations in saliva using low-cost thin-layer chromatography (TLC) and nevirapine concentrations in plasma and saliva using high performance liquid chromatography (HPLC) methods; and to correlate nevirapine plasma concentrations to HIV treatment outcomes in Ugandan patients.

**Methods:**

Paired plasma and stimulated saliva samples were obtained from Ugandan, HIV-infected adults on nevirapine-based ART. Nevirapine concentrations were measured using a validated HPLC method and a novel TLC method. Plasma nevirapine concentrations <3.0 mg/L using HPLC were considered subtherapeutic. Negative/positive predictive values of different thresholds for subtherapeutic nevirapine concentrations in saliva were determined. Virologic testing and, if applicable, HIV drug resistance testing was performed.

**Results:**

Median (interquartile range, IQR) age of 297 patients was 39.1 (32.8-45.2) years. Three hundred saliva and 287 plasma samples were available for analysis. Attempts failed to determine nevirapine saliva concentrations by TLC. Using HPLC, median (IQR) nevirapine concentrations in saliva and plasma were 3.40 (2.59-4.47) mg/L and 6.17 (4.79-7.96) mg/L, respectively. The mean (coefficient of variation,%) nevirapine saliva/plasma ratio was 0.58 (62%). A cut-off value of 1.60 mg/L nevirapine in saliva was associated with a negative/positive predictive value of 0.99/0.72 and a sensitivity/specificity of 87%/98% for predicting subtherapeutic nevirapine plasma concentrations, respectively. Only 5% (15/287) of patients had subtherapeutic nevirapine plasma concentrations, of which 3 patients had viral load results > 400 copies/mL. Patients with nevirapine concentrations in plasma <3.0 mg/L had an Odds Ratio of 3.29 (95% CI: 1.00 – 10.74) for virological failure (viral load >400 copies/mL).

**Conclusions:**

The low-cost TLC technique for monitoring nevirapine in saliva was unsuccessful but monitoring nevirapine saliva and plasma concentrations using HPLC was shown to be feasible in the research/specialist context in Uganda. Further optimization and validation is required for the low-cost TLC technique.

## Background

Access to antiretroviral therapy (ART) is improving in developing countries which experience the greatest disease burden arising from HIV infection. The successful roll-out of ART was achieved by adopting a public health approach to HIV care and treatment involving cost minimization strategies, delegation of tasks from highly skilled to less skilled health workers and simplification of the routine laboratory tests that are used to monitor ongoing efficacy of ART [1, 2]. Due to cost constraints, laboratory monitoring of ART efficacy is often limited to the CD4 cell count - a test that has low accuracy for identifying patients experiencing treatment failure to ART [3]. The gold standard for treatment failure, viral load testing, is largely unavailable because of its complexity and costs. Low-cost laboratory tests are needed to optimize ART monitoring in developing countries.

Nevirapine, a non-nucleoside reverse transcriptase inhibitor, is one of the most widely used components of first-line ART, because of its low cost, good long-term tolerability, and high efficacy [4, 5]. However, a disadvantage of nevirapine is the emergence of drug resistant virus due to its long elimination half-life and low genetic barrier to resistance, as one single resistance mutation can result in complete loss of virologic efficacy [6]. Notably, suboptimal plasma concentrations of antiretroviral drugs may increase the risk of treatment failure [7]. For therapeutic drug monitoring (TDM) of nevirapine a trough plasma concentration >3.0 mg/L is suggested [8, 9]. However, nevirapine pharmacokinetics exhibit marked interpatient variability that can be explained in part by genetic differences, drug-drug interactions, pregnancy and other (ethnic) factors [10–12].

Adherence to ART is critical for treatment success, but routine clinical measures of adherence (e.g. patient self-report) are subjective and can be prone to social desirability bias. To date, there are no recommended laboratory tests to directly evaluate adherence to ART in developing countries [13, 14]. Confirmation of treatment failure is limited by the high cost of definitive diagnostics (e.g. HIV viral load and resistance testing). Consequently, in resource-limited settings, it is often difficult to distinguish patients who are non-adherent from patients with treatment failure arising from the emergence of drug-resistant virus. International guidelines recommend that patients with suspected treatment failure to nevirapine-based ART are switched to second-line regimens being significantly more expensive than first-line regimens [2, 15]. Thus, TDM can be used to identify non-adherent patients and thereby prevent unnecessary switching to more expensive ART regimens [16, 17].

However, TDM can only be recommended if such methods are accurate, robust, available and affordable in developing countries. To this end, one African study validated the use of a TLC technique as a low cost alternative to HPLC for TDM in patients using nevirapine [18]. Nevirapine concentrations are generally measured in blood samples, but saliva has been reported to be an acceptable alternative matrix [19, 20]. Saliva sampling is less invasive than blood sampling and so it may be preferable for certain special populations (e.g. children). However, clinical experience is limited and therapeutic cut-offs for nevirapine in saliva have not yet been evaluated in relation to virologic failures.

This study aimed to validate nevirapine concentration measurements in saliva samples using TLC and nevirapine concentrations in saliva and plasma using standard validated HPLC method. Furthermore, we aimed to correlate subtherapeutic nevirapine levels with virological outcome of ART and HIV drug resistance development in a cohort of Ugandan adults on (long-term) ART.

## Methods

This was a cross-sectional pharmacokinetic study, which was conducted at three clinical sites (Mbale, Fort Portal and Kampala) of the Joint Clinical Research Centre - a major provider of ART in Uganda. The study was designed as a sub-study within a longitudinal prospective antiretroviral monitoring study: the Pan African Studies to Evaluate Resistance (PASER) program (http://aighd.org/projects/paser/). Ethics approval was obtained from the Joint Clinical Research Clinic (JCRC) Institutional Review Board, Kampala, Uganda and the Academic Medical Center Institutional Review Board, Amsterdam, The Netherlands. Written informed consent was obtained from all patients prior to participation in the study.

Participants were HIV-1 infected adults receiving nevirapine as part of their ART regimen for at least 24 months. In order to reach the target sample size of 300 patients, additional non-PASER patients attending ART clinics at the three study sites were recruited. The study excluded patients with oral lesions or ulcers or serious illness requiring immediate treatment or hospitalization. With a sample size of 300 subjects and assuming a 10% rate of sub-therapeutic values, the proportion of patients with sub-therapeutic nevirapine concentrations in saliva and plasma could be measured with a 95% confidence interval of 7.4% to 12.6%.

Study patients underwent a full clinical assessment including collection of demographic data, WHO clinical stage and self-reported adherence in the last 3 and 30 days before sampling [21]. Routine laboratory results including CD4 cell count, viral load and hemoglobin were obtained from local laboratory records. Prior to pharmacokinetic blood and saliva sampling, participants provided information on the time of last intake of nevirapine and use of any concomitant medicines over the preceding 24 hours.

Venous whole blood samples were obtained from study participants and EDTA anti-coagulated plasma specimens were stored at −80°C. Saliva samples were obtained using a dental cotton roll impregnated with citric acid (Salivette®, Sarstedt, Etten-Leur, The Netherlands). From each patient, approximately 5 mL of saliva was isolated by centrifugation of the cotton roll. Two aliquots of each saliva sample were stored at −80°C.

Nevirapine concentrations were measured in paired plasma and saliva samples using a validated HPLC method at the Infectious Diseases Institute, Kampala, Uganda (lower limit of quantification 0.05 mg/L) [18]. The accuracy of the HPLC assay ranged from 94 – 96% for saliva and 94 – 99% for plasma. Individual and mean nevirapine saliva/plasma ratios were determined. Plasma nevirapine concentrations below 3.0 mg/L were considered sub-therapeutic.

Nevirapine concentrations in saliva samples were also measured at the JCRC reference laboratory in Kampala, Uganda using an earlier described TLC technique which reported a sensitivity of 92% and specificity of 99% when compared to plasma concentrations determined by HPLC [18]. For HPLC measurements, we determined the negative and positive predictive values of different thresholds for sub-therapeutic saliva concentrations of nevirapine for predicting nevirapine plasma concentrations above or below 3.0 mg/L.

HIV-RNA determination and genotypic resistance testing if HIV-RNA was >1000 copies/mL was performed, as described elsewhere [22]. Drug resistance mutations were scored according to the 2013 International AIDS Society-USA list [23]. Subtypes were determined using the SCUEAL HIV-1 subtyping tool [24] and additional analysis with the REGA algorithm version 2.0 [25], if required.

Clinical and demographic data were summarized and were presented as median values with interquartile ranges and numbers percentages for categorical values.

## Results

A total of 297 patients were enrolled in the study: 120 (40.4%) were co-enrolled from the PASER-M cohort and the remaining 177 (59.6%) were enrolled from antiretroviral clinics in the three study sites. Demographic characteristics are shown in Table 1.

**Table 1:** **Baseline characteristics of study patients**

Characteristic	Total (n = 297)	Nevirapine plasma conc. ≥3.0 mg/L (n = 282)	Nevirapine plasma conc. <3.0 mg/L (n = 15)
Sex, female	201 (67.7)	192 (68.1)	9 (60.0)
Age (years)	39.1 (32.8-45.2)	39.2 (33.1-45.2)	33.3 (30.9-42.8)
Time on ART (months)	28.5 (25.8-33.5)	28.1 (25.9-32.6)	31.1 (24.4-41.2)
NRTI backbone			
zidovudine	235 (79.1)	223 (79.1)	12 (80.0)
tenofovir	19 (6.4)	17 (6.0)	2 (13.3)
HIV-RNA >400 copies/mL*	21 (8.9)	18 (8.1)	3 (23.1)
30-day adherence			
100%	260 (91.6)	248 (91.9)	12 (85.7)
95- < 100%	19 (6.7)	18 (6.7)	1 (7.1)
<95%	5 (1.8)	4 (1.5)	1 (7.1)
3-day adherence (any pills missed)	9 (3.3)	6 (2.3)	3 (20.0)
CD4 cell count (cells/mm^3^)	363 (265–509)	385 (269.5-511)	299 (211–354)

Baseline characteristics stratified by patients with therapeutic (≥3.0 mg/l) or subtherapeutic (<3.0 mg/L) nevirapine plasma concentrations.

Values in brackets are n (%) for categorical variables and median (interquartile range, IQR) for continuous variables. * HIV-RNA results from 235 patients were available.

Two hundred and ninety seven saliva samples and 287 plasma samples were available for analysis. Median (IQR) nevirapine concentrations in saliva and plasma was 3.40 (2.59-4.47) mg/L and 6.17 (4.79-7.96) mg/L, respectively. Corresponding mean (standard deviation) concentrations for saliva and plasma were 6.71 (3.39) and 3.72 (2.16) mg/L, respectively.

We found a strong positive correlation between nevirapine concentrations in plasma and saliva (Spearman’s rho, 0.886, *p* <0.001).

Only 15 patients (5%) had nevirapine plasma concentrations below 3.0 mg/L by using HPLC. The mean (coefficient of variation,%) nevirapine saliva-to-plasma ratio was 0.58 (62%). A cut-off nevirapine concentration of 1.60 mg/L in saliva was associated with the highest negative and positive predicted values of 0.99 and 0.72, respectively and with the highest sensitivity and specificity of 87% and 98%, respectively for predicting nevirapine plasma concentrations below 3.0 mg/L. Area under the receiver operating characteristic curve was 0.924 (95% confidence interval (CI), 0.83 - 1.00).

Despite multiple attempts, it was not possible to determine nevirapine concentrations in saliva samples following the TLC technique used by L’homme *et al.* in Tanzania [18]. There were no nevirapine spots detectable on the TLC plates or spots from the validated by HPLC nevirapine reference solutions. Structured trouble-shooting by following a step-by-step procedure to locate the error in the assay was unsuccessful. Therefore, analysis using the TLC method was terminated from the current study.

As stated, 5% (15/287) of study patients had sub-therapeutic nevirapine plasma concentrations. Thirteen of these patients had a viral load result available and 3 patients (23%) had a viral load result of > 400 copies/mL, see Table 2. Patients with sub-therapeutic nevirapine concentrations in plasma (<3.0 mg/L) had an Odds Ratio (OR) of 3.29 (95% CI: 1.00 – 10.74) for virological failure (viral load >400 copies/mL) than patients with adequate nevirapine plasma concentrations. Among the 21 patients with viral load >400 copies/mL, genotypic resistance mutations were detected in 14 out of 15 patients with available genotyping results. Only 2 of these patients had sub-therapeutic nevirapine concentrations in plasma (Table 2). The most common nevirapine-associated HIV drug resistance mutations were K103N, Y181C and G190A.

**Table 2:** **Characteristics of patients with virological failure (HIV-RNA >400 copies/mL) and nevirapine plasma concentrations below 3 mg/L**

Patient ID	sex	Age (years)	Nevirapine plasma conc. (mg/L)	30-day adherence	Viral load (copies/mL)	NNRTI DRMs	NRTI DRMs
MBA029	female	30	0.44	100%	40768	K103N	M184IMV
JCR089	female	32	0.21	95- < 100%	966	ND	ND
JCR086	male	48	0.03	100%	696298	G190A	None

NNRTI = non-nucleoside reverse transcriptase inhibitor, NRTI = nucleoside reverse transcriptase inhibitor, DRM = drug resistance mutation (as identified Stanford Genotypic Resistance Interpretation Algorithm), ND = no data available.

## Discussion

Using HPLC, monitoring nevirapine concentrations in saliva and plasma samples was shown to be feasible and potentially useful in a specialist and research facility in Uganda. In contrast, the low-cost TLC technique did not appear robust. For unknown reasons, in Uganda, attempts to set-up the TLC assays for nevirapine in saliva failed, while in neighbouring Tanzania the same method was successful and validated [18]. In other studies, the TLC technique was successful for the semi-quantitative determination of nevirapine in saliva and other matrices [26–29]. In these studies, the low-cost TLC method was reported as sensitive, specific, robust, and able to detect sub-therapeutic concentrations of the drug, and the results compared well with HPLC.

However, the TLC method suffers from two key shortcomings: the reliance on the laboratory technicians’ visual acuity for estimation of drug content, and the lab technicians’ skills for manually applying sample solution spots on the chromatographic plate with adequate precision [30]. In our study, the laboratory technicians were well trained for this procedure and no definite cause was established for the failure of the TLC technique in the present study. Hypotheses to explain the failure of the nevirapine TLC assay include the influence of local temperature and humidity on the TLC plates that were stored at the site approximately 18 months during the conduct of the study.

As determined by HPLC, nevirapine concentrations in plasma were within the expected range and the calculated nevirapine saliva-to-plasma ratio of 0.58 was consistent with previous reports [18, 21]. Factors influencing the penetration of drugs into saliva include molecular size, plasma protein binding, saliva flow rates and lipid solubility of drugs [31]. Indeed, approximately 60% of nevirapine in plasma is bound to plasma proteins and the free fraction of nevirapine could drive significant quantities of nevirapine into saliva and other matrices. For instance, a cerebrospinal fluid to plasma ratio of 0.45 has been reported for nevirapine [32].

We determined the cut-off value for nevirapine in saliva was 1.60 mg/L with a high positive and negative predictive value for identifying a patient with a sub-therapeutic nevirapine concentration in plasma. Previously, cut-offs of 1.5 mg/L and 1.75 mg/L have been used [13, 29].

Although patients with sub-therapeutic concentrations of nevirapine had increased odds of having a viral load > 400 copies/mL, the magnitude of odds ratio was small and the lower bounds of the 95% confidence interval included the no-effect boundary of 1. In contrast, an observational cohort analysis in Netherlands which followed up 189 patients and obtained untimed nevirapine plasma samples at approximately 6 months after commencing nevirapine-based ART, found that subtherapeutic nevirapine concentrations were associated with increased risk of subsequent virologic failure (relative risk 5.0 95% CI, 1.8 – 13.7).

Due to the cross-sectional nature of the current study that was conducted at 2 years after commencement of ART, the study population could be prone to selection bias arising from drop-outs within the first two years of ART. It is conceivable that prior to the second year of ART, some patients with sub-therapeutic concentrations could experience treatment failure and switch therapy, experience opportunistic infections that would preclude them from study participation or die. Importantly, such a scenario would likely bias towards the null an association between sub-therapeutic concentrations and viral loads >400 copies/mL. In the current study, the proportion of patients with sub-therapeutic nevirapine concentrations was 5%, which was lower than the estimate of 10% which was utilized in sample size calculations. In two Tanzanian studies, sub-therapeutic nevirapine concentrations in plasma were observed in 9% [18] and 13.2% [29] of samples. Consequently, a Type II error in the current study cannot be ruled out.

An important study limitation is that adherence was obtained by patient self-report and it is possible that this was overestimated, particularly among subjects with negligible nevirapine concentrations. Furthermore, the study was cross-sectional in design and as such temporal variation in adherence over time was not captured. This limitation could have resulted in the poor correlation of adherence to virologic failure as it is possible that previously non-adherent patients who develop virologic failure could subsequently improve on their adherence. Moreover, this study design cannot be used to determine the effectiveness of TDM. Instead, prospective, randomized studies in resource limited settings should be considered to address this question.

Due to financial and technical constraints monitoring conventional viral load and HIV-drug resistance testing is infrequently performed in resource-limited settings. However, recent technological advances are likely to enable lower test costs, to simplify sample storage using dried blood spots and to utilize equipment that require less technical expertise [33]. These novel tests are currently being piloted at various locations in sub-Saharan Africa. In developing countries, non-invasive saliva sampling, in combination with the use of a robust and low cost assay could complement viral load testing and potentially become an attractive alternative to resistance testing among patients who are suspected to be primarily non-adherent. Our findings suggest that the low-cost TLC technique may not be sufficiently robust for widespread implementation. In contrast, the HPLC assay was shown to be robust in a resource-limited setting. Furthermore, the availability of HPLC in countries like Uganda would diminish the need for TLC methodology. However, when compared to virologic monitoring, it is unlikely that the HPLC monitoring would be cost-effective in these settings given the high set-up costs and the limited clinical role of results from pharmacologic tests.

## Conclusions

In conclusion, while the low-cost TLC technique for monitoring nevirapine in saliva was unsuccessful, monitoring nevirapine saliva and plasma concentrations using HPLC was feasible in a specialist and research centre in Uganda. While HPLC technology may not be adaptable for widespread use in resource-limited settings, investment in this technology may be justified in specialist centres that provide support to large ART clinics or multiple clinical sites. Further optimization and validation is required for the low cost TLC technique.
